# A New Spirit in the Medical Profession of Georgia

**Published:** 1871-12

**Authors:** 


### EDITORIAL AND MISCELLANEOUS.

### A NEW SPIRIT IN THE MEDICAL PROFESSION OF GEORGIA.

We are constantly receiving evidences that a new spirit is being aroused in the medical profession of Georgia. Now that the deadly upas of strife has been plucked up by the roots, we are beginning to see clearly the dawn of a better day. Many of our best men have had their interest in the general welfare of the profession completely paralyzed by the controversies which have disturbed their chief organization for a number of years, and, as the result, they had discontinued their attendance upon its annual meeting; but with the assurance furnished by the action of the last meeting of the the Georgia Medical Association, that these elements of discord will not be allowed another entrance into that body, we shall have not only a much larger attendance upon its deliberations, but the presence of those who go in the interests of science and humanity, prepared to contribute valuable papers to its deliberations.

We hope and believe that this journal is doing something towards arousing some of our best medical men from the lethargy which a majority of them have indulged during and since the war—at least, so far as any contribution for the general advance of the profession is concerned. It is very certain that we have a large amount of the highest order of ability in the medical profession of this State; and we are confident that, after the next meeting of the Georgia Medical Association, at Columbus, in April, the transactions will no longer bear an odious comparison with those of our sister States of Alabama and South Carolina.

